# Deteriorative Effects of Radiation Injury Combined with Skin Wounding in a Mouse Model

**DOI:** 10.3390/toxics10120785

**Published:** 2022-12-14

**Authors:** Li Wang, Bin Lin, Min Zhai, Wanchang Cui, Lisa Hull, Alex Zizzo, Xianghong Li, Juliann G. Kiang, Mang Xiao

**Affiliations:** 1 Radiation Combined Injury Program, Armed Forces Radiobiology Research Institute, Uniformed Services University of the Health Sciences, Bethesda, MD 20814, USA; 2 Henry M. Jackson Foundation for the Advancement of Military Medicine, Inc., Bethesda, MD 20817, USA; 3 Department of Pathology, Uniformed Services University of the Health Sciences, Bethesda, MD 20814, USA; 4 Department of Pharmacology and Molecular Therapeutics, Uniformed Services University of the Health Sciences, Bethesda, MD 20814, USA; 5 Department of Medicine, Uniformed Services University of the Health Sciences, Bethesda, MD 20814, USA

**Keywords:** radiation-combined injury, skin wounding, delayed wound healing, systemic proinflammatory status, hematopoietic suppression, cytokines and chemokines, growth factors, dysregulated collagen homeostasis

## Abstract

Radiation-combined injury (RCI) augments the risk of morbidity and mortality when compared to radiation injury (RI) alone. No FDA-approved medical countermeasures (MCMs) are available for treating RCI. Previous studies implied that RI and RCI elicit differential mechanisms leading to their detrimental effects. We hypothesize that accelerating wound healing improves the survival of RCI mice. In the current study, we examined the effects of RCI at different doses on lethality, weight loss, wound closure delay, and proinflammatory status, and assessed the relative contribution of systemic and local elements to their delayed wound closure. Our data demonstrated that RCI increased the lethality and weight loss, delayed skin wound closure, and induced a systemic proinflammatory status in a radiation dose-dependent manner. We also demonstrated that delayed wound closure did not specifically depend on the extent of hematopoietic suppression, but was significantly influenced by the toxicity of the radiation-induced systemic inflammation and local elements, including the altered levels of proinflammatory chemokines and factors, and the dysregulated collagen homeostasis in the wounded area. In conclusion, the results from our study indicate a close association between delayed wound healing and the significantly altered pathways in RCI mice. This insightful information may contribute to the evaluation of the prognosis of RCI and development of MCMs for RCI.

## 1. Introduction

Radiation-combined injury (RCI) is the ionizing radiation exposure combined with physical trauma, such as skin wound or burn, chemical trauma, infection, and/or physiological insults [1,2]. RCI is predicted to comprise more than 60% of injuries in a mass casualty incident. Therefore, it would be anticipated during a radiation public health emergency involving a detonation. [1]. In general, RCI patients have a worse overall prognosis than do patients with radiation injury (RI) alone or trauma alone, because the healing of wounds/fractures is significantly delayed, infections are much more difficult to control, and/or chances of survival after RCI are lower than after RI alone [1,3,4,5,6]. Increased morbidity and mortality in RCI are synergistically affected by radiation exposure and combined trauma [7]. For example, in the combined injury of radiation exposure followed by a full-thickness skin wounding, both radiation and wounding not only induce a similar physiological responses (e.g., fluid loss, metabolic changes, or shock with changes in hormone concentration) but also cause damage to cellular compartments (e.g., bone marrow, gastrointestinal tract, and skin), resulting in impaired wound repair, poor tissue recovery, immunosuppression, increased susceptibility to microbial infections, sepsis, and lethality [4,7,8,9].

Even though RCI is more frequent and causes more severe symptoms compared to RI alone in a mass casualty incident, no United States Food and Drug Administration (US FDA)-approved medical countermeasures (MCMs) are available for mitigating/treating RCI. To improve the preparedness for attacks using radiation devices of mass destruction, and, particularly, to promote the development of MCMs for mitigating/treating RCI, several animal models for different types of RCI have been established [1]. At the Armed Forces Radiobiology Research Institute (AFRRI), a mouse model of total body irradiation (TBI), combined with a full-thickness, non-lethal skin wound (SW), was developed and has been extensively used in screening potential MCMs against RCI [5,7,10,11,12,13,14,15,16,17,18,19,20,21]. Studies using this mouse model first demonstrated that RCI dramatically increases the 30-day mortality and decreases radiation LD_50/30_ (lethal dose of radiation anticipated to cause 50% of deaths of the irradiated animal within 30 days) level compared to RI alone [4,7], and, RCI mice die earlier than RI mice. A pattern that shows 9.5/9.75 Gy RCI delaying the wound closure by one to two weeks compared to SW alone is also commonly observed [4,19]. Multiple studies conducted for MCM screening have demonstrated that leukocyte growth factors, US FDA-approved mitigators for hematopoietic acute radiation syndrome (H-ARS), such as the granulocyte-colony stimulating factor (G-CSF) and PEGylated granulocyte colony-stimulating factor (PEG-G-CSF) [16,22,23,24], fail to enhance the survival of mice after RCI [16,21], possibly because they further delay wound closure in RCI animals compared to the vehicle treatment [15]. However, other products that have been tested in our RCI mouse model, such as ghrelin [17], ciprofloxacin [10], L-citrulline [21], and bone marrow mesenchymal stem cells (BMSCs) [12], mitigate RCI, at least partially, possibly due to their effects on accelerating wound closure in RCI animals.

Due to the synergistic impact between radiation exposure and the combined trauma, it appears that mechanisms underlying the deteriorative effects of RCI are more complicated than those of the individual injury (e.g., RI alone or SW alone) [13]. It was proposed that the delayed wound closure and related complications such as infection and sepsis are the leading causes of early death after RCI [19]. Normal wound healing is achieved by four well-defined phases (hemostasis, inflammation, proliferation, and remodeling) occurring in the right order and time span [25,26]. There are local and systemic factors that can impact wound healing by interfering with one or more highly programmed phases in the normal healing process, thus leading to the delayed wound healing [25]. Factors local to the wound itself have a direct impact on the characteristics of the wound, whereas the systemic factors affect the wound healing capability and many of them act through local factors [25]. It was suggested that, compared to local skin irradiation, the TBI induced significantly less infiltration of the inflammatory cells in full-thickness skin wounds, most likely resulting from the TBI-mediated hematopoietic suppression [27]. The TBI-induced decrease in inflammatory cell content might reduce chemokine production in skin wounds [28], thereby delaying wound closure.

This study aimed to examine the effects of RCI on skin-wound healing and understand the mechanisms by which RCI increased the risk of mortality in animals. We compared animal lethality, body weight, wound healing, and systemic/local inflammatory status after RCI at different TBI doses (8.5/9.0/9.5 Gy) and identified parameters affecting wound healing in animals subjected to SW alone or RCI.

## 2. Materials and Methods

### 2.1. Mice

A RCI mouse model has been established in our laboratory using female B6D2F1/J mice. Animals were obtained from the Jackson Laboratory (Bar Harbor, ME, USA) and were 13–15 weeks old at time of use. The reason we chose female mice is that male mice are more likely to sustain unnecessary injuries because they are more aggressive when housed together, as described [21]. All mice were required to acclimate to their new surroundings for at least 72 h after arrival. We randomly assigned mice to experimental groups, and 5 mice per cage were housed in a facility accredited by the Association for Assessment and Accreditation of Laboratory Animal Care, International (AAALAC International). The animal room followed a 12:12 h light-dark schedule, and was also properly maintained at 23 °C ± 3 °C and 50% ± 20% relative humidity. To all mice, acidified water (pH 2.5–3.0) and commercial rodent feed (Envigo Teklad Rodent Diet; Envigo Inc. Indianapolis, IN, USA) were available ad libitum. All animal handling procedures were performed in compliance with the guidelines from the National Research Council (2011), and animal studies were conducted according to a protocol approved by the Institutional Animal Care and Use Committee (IACUC) at the Uniformed Services University of the Health Sciences (USUHS, Bethesda, MD, USA).

### 2.2. Total Body Irradiation

Female mice, 24–25 g of average body weight and around 14–15 weeks old, were exposed to total body irradiation (TBI) from a bilateral radiation field at AFRRI’s ^60^Co facility, as previously described [21]. To determine dose rates (to water) in the cores of acrylic mouse phantoms, we employed an alanine/electron spin resonance (ESR) dosimetry system (American Society for Testing and Materials, Standard E 1607; ASTI International, Philadelphia, PA, USA). Before TBI, we placed 40 mice in ventilated plexiglass containers (four mice each container with separate compartments for each animal), which were irradiated at once without anesthesia. The distance between the irradiators and animals determines the dose rate. A single, mid-line tissue dose of 8.5 Gy, 9.0 Gy, and 9.5 Gy was delivered at approximately 0.4 Gy/min. Animals in sham and skin wounding (SW) alone groups were handled in the same manner as the irradiated animals but remained in the Co-60 facility staging room.

### 2.3. Skin Wounding

The wounding procedure comprises 2 steps, the skin shaving and skin wounding, both of which were conducted under anesthesia by isoflurane inhalation, as described [21]. To prepare for wounding, the dorsal fur was shaved using an electric hair clipper two-three days prior to TBI. Within 1–2 h following the sham irradiation or TBI, a 250–300-mm^2^ circular-like full thickness skin injury was created in the anterior-dorsal skin fold and the underlying panniculus carnosus muscle (between the shoulder blades) by a 70% ethanol-sterilized steel punch for animals in SW alone and RCI groups. After the skin injury, all mice were placed in autoclaved clean cages containing autoclaved bedding and these non-lethal wounds were left open to the environment. To alleviate the acute pain and minimize distress, all mice subjected to the skin injury were intraperitoneally (I.P.) administrated 0.5 mL of acetaminophen solution (150 mg/kg diluted in saline, OFIRMEV injection, NDC 43825-102-01; Mallinckrodte Pharmaceuticals, Hazelwood, MO, USA), immediately after skin wounding, following the 3 Rs policy (refinement, reduction, and replacement) of the USUHS IACUC. Sham animals were also anesthetized, shaved, and then handled without skin wounding. In addition, 0.5 mL saline was administered I.P. to the sham group as a control.

### 2.4. Thirty-Day Mice Survival

Researchers closely monitored mice for 30 days after RCI or SW, in addition to the regular health checks by vivarium staff. During the 30 days, we strictly followed the USUHS IACUC Policy 020 (establishment of early endpoints in a mouse TBI model) [29]. Morbid animals were checked at least three times daily, and the moribund animals were euthanized by CO_2_ inhalation plus confirmatory cervical dislocation. We recorded the percentage of mice surviving through the entire 30 days, and plotted the data using the Kaplan-Meier survival curve.

### 2.5. Body Weight and Wound Measurements

The body weight was measured immediately following TBI (on day 0, referred to as basal body weight), and on days 1, 3, 7, 14, 21, and 28 post-TBI. We calculated the percentage (%) of body weight as % body weight = 100% * body weight measured on day X/basal body weight, day X: days 1, 3, 7, 14, 21, and 28 post-TBI. Wound size was assessed using a digital caliper on days 1 (referred to as basal wound size), 3, 7, 14, 21, and 28 post-TBI. We calculated the average area of each wound according to previously reported work [4]: Wound area = π * A/2 * B/2 (A and B represent diameters at right angles to each other, and A is defined as the maximal length of a given shape). The percentage of wound closure was then calculated as % wound closure = 100% - (wound area on day X/wound area on day 1) * 100%; day X: days 3, 7, 14, 21, and 28 post-TBI. A 100% wound closure specified a fully-closed wound, as described [21]. In addition, the time to reach a completed wound closure was recorded and plotted using the Kaplan-Meier curve.

### 2.6. Blood Collection, Peripheral Blood Cell Count, Serum Preparation, and Tissue Collection

Under deep isoflurane anesthesia, blood was drawn from the 30-day surviving mice via cardiac puncture into a microtube containing EDTA for complete blood count (CBC), and into a microtube with a serum separator additive for serum preparation, as described [30]. Whole blood samples, at least 15 µL, were analyzed using a veterinary hematology analyzer (Element HT5; Heska, Loveland, CO, USA) following the manufacturer’s instructions. Sera were collected after centrifugation at 10,000× *g* for 10 min following at least 30 min coagulation at room temperature (RT), which were immediately stored at –80 °C for further analysis. After the blood draw, cervical dislocation was performed, and then the skin and sternum were collected for further analysis.

### 2.7. Quantified Cytokine Array: Serum and Skin Tissue Lysate

Serum samples for cytokine array were prepared as described above. The frozen mouse skin tissues were homogenized in radioimmunoprecipitation assay (RIPA) lysis and extract buffer (Thermo Scientific, Rockford, IL, USA) supplemented with a Halt^TM^ protease inhibitor Cocktail (Thermo Scientific, Rockford, IL, USA) by Stomachert 80 Biomaster Lab System (Seward Laboratory Systems, Port St. Lucie, FL, USA) following the manufacturer recommendations. Skin lysates (the supernatants) were collected after centrifugation at 12,000 g/min for 15-min, and protein concentrations of skin lysates were determined using a bicinchoninic acid (BCA) protein assay kit (Thermo Scientific, Rockford, IL). Serum and skin tissue lysates were subjected to a quantified mouse cytokine array analysis by Eve Technologies Company (Calgary, AB, Canada). In brief, 100 µL of the mouse serum at a 2-fold dilution with PBS pH 7.4 and skin tissue lysate at the concentration of 4 µg/µL was subjected to a mouse cytokine/chemokine 44-plex discovery assay^®^ array (MD44). A total of 44 cytokines measured, included eotaxin, erythropoietin, 6Ckine, fractalkine, G-CSF, GM-CSF, IFNB1, IFNγ, IL-1α, IL-1β, IL-2, IL-3, IL-4, IL-5, IL-6, IL-7, IL-9, IL-10, IL-11, IL-12p40, IL-12p70, IL-13, IL-15, IL-16, IL-17, IL-20, IP-10, KC, LIF, LIX, MCP-1, MCP-5, M-CSF, MDC, MIG, MIP-1α, MIP-1β, MIP-2, MIP-3α, MIP-3B, RANTES, TARC, TIMP-1, TNFα, and VEGF-A.

### 2.8. Western Blot Analysis

Skin tissue lysate (20–30 μg) was loaded into NuPAGE 4–12% Bis-Tris gels (Invitrogen, Waltham, MA) and separated by sodium dodecyl-sulfate polyacrylamide gel electrophoresis (SDS-PAGE). Two primary antibodies were used: rabbit (Rb) anti-connective tissue growth factor (CTGF, 1:500 dilution, Catalog # orb10474, Biorbyt, Cambridge, CB4 0WY, UK), and mouse anti-Vinculin (2 µg/mL, Catalog # MAB6896, R&D Systems, Minneapolis, MN, USA). Horseradish peroxidase (HRP)-conjugated anti-Rb and anti-mouse (Ms) antibodies were employed as secondary antibodies (R&D Systems, Minneapolis, MN, USA) following the manufacturer’s instructions. Protein expression was visualized with Western ECL Blotting Substrates (ThermoFisher, Waltham, MA, USA), and images were acquired using a Bio-Rad ChemiDoc MP Imaging System. Densitometry was analyzed using Image Lab (Version 6.0.1, Standard Edition, Bio-Rad Laboratories, Inc.). Vinculin served as a loading control [31] because its expression in the skin tissue is not affected by radiation/wounding.

### 2.9. Histological Examination of Skin and Sternum

Tissues including sternum and skin around the wounds harvested from the 30-day surviving animals were immediately fixed in 10% formalin for at least 24 h, washed with PBS, and then stored in 70% ethanol at room temperature. For histological examination, dehydration, paraffin embedding, sectioning into 5-µm-thick cross sections, and then hematoxylin and eosin (H&E) staining were performed as described [21]. Bones (sternums) were decalcified before paraffin sectioning. To assess the collagen deposition, mouse skin sections were stained using Masson’s trichrome staining (Catalog # 25088-1; Polysciences; Warrington, PA, USA) following the manufacturer’s instruction. To evaluate the activity of the myofibroblast, immunohistochemical (IHC) staining of alpha-smooth muscle actin (α-SMA) in mouse skin sections was performed as described [32]. In brief, sections were incubated with Rb anti-α-SMA (1:1000 dilution, catalog # 124964, Abcam; Cambridge, MA, USA) overnight at 4 °C. Following extensive washes with PBS, sections were then incubated with anti-Rb secondary antibody conjugated with Alexa Fluor 568 (ThemoFisher; Waltham, MA, USA) for one hour at RT and mounted with a Prolong antifade mounting medium with DAPI (ThemoFisher; Waltham, MA, USA). The bright-field and fluorescent images of stained slides were obtained using the Zeiss Axioscan.Z1 and analyzed using Zeiss Zen 2.5 (blue edition) (Carl Zeiss AG, Oberkochen, Germany).

### 2.10. Statistical Analysis

We performed data analysis using GraphPad Prism version 7 (La Jolla, CA, USA), and expressed the results as mean ± standard error of the mean (SEM). Log-rank (Mantel-Cox) test was employed to compare the Kaplan-Meier curves. The difference among groups was analyzed by either one-way or two-way analysis of variance (ANOVA) with Dunnett’s or Tukey’s multiple comparisons, Mann–Whitney Test (unpaired, two-tailed), or unpaired *t*-test (two-tailed). *p* < 0.05 was considered statistically significant.

## 3. Results

### 3.1. RCI Results in Increases in 30-Day Lethality, Body Weight Loss, and Delayed Wound Closure in a TBI Dose-Dependent Manner

**Figure 1:**
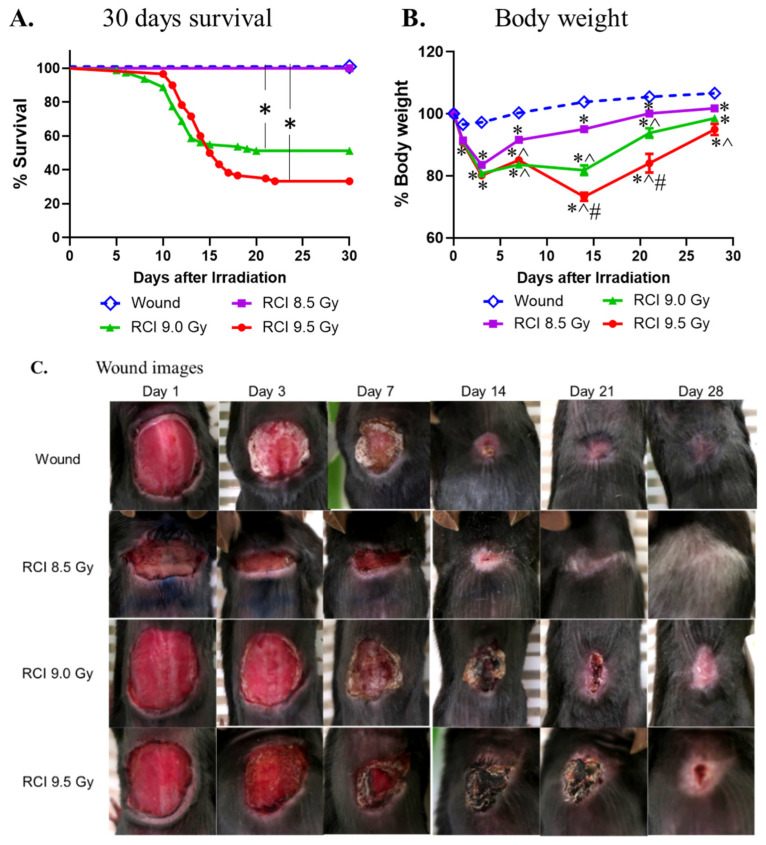

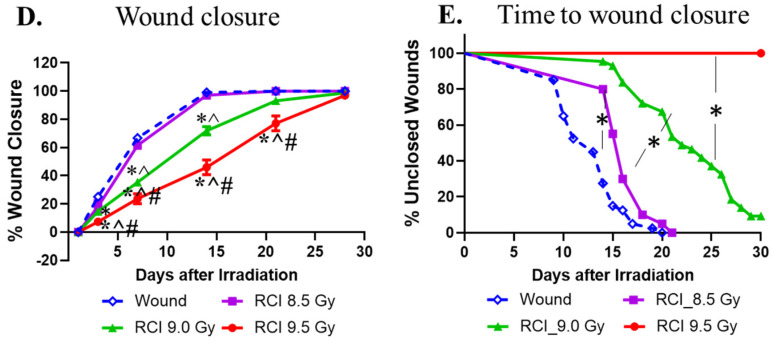
The effects of RCI doses on 30-day lethality, body weight loss, and wound closure delay. (**A**) The 8.5 Gy is a sub-lethal dose, and 9.0 Gy and 9.5 Gy are lethal doses for RCI. * *p* < 0.05 for Wound/RCI 8.5 Gy vs. 9.0 Gy and Wound/8.5 Gy vs. 9.5 Gy by Log-rank (Mantel-Cox) test; N = 80/20/80/60 for Wound/RCI 8.5/9.0/9.5 Gy. (**B**) RCI resulted in significantly dose-dependent body weight loss. * *p* < 0.05, vs. Wound; ^ *p* < 0.05, vs. RCI 8.5 Gy; # *p* < 0.05, vs. RCI 9.0 Gy by two-way ANOVA with Tukey’s multiple comparisons tests; N = 80/20/80/60 for wound/RCI 8.5/9.0/9.5 Gy. (**C**) Representative images of the wounds from Wound, RCI at 8.5 Gy, RCI at 9.0 Gy, and RCI at 9.5 Gy groups on days 1, 3, 7, 14, 21, and 28 after TBI. (**D**) RCI delayed wound closure in a dose-dependent fashion. * *p* < 0.05, vs. Wound; ^ *p* < 0.05, vs. RCI 8.5 Gy; # *p* < 0.05, vs. RCI 9.0 Gy by two-way ANOVA with Tukey’s multiple comparisons tests; N = 80/20/80/60 for wound/RCI 8.5/9.0/9.5 Gy. (**E**) RCI dose-dependently extended the time to wound closure. * *p* < 0.05 for wound vs. RCI 8.5 Gy, RCI 8.5 Gy vs. RCI 9.0 Gy, and RCI 9.0 Gy vs. RCI 9.5 Gy by Log-rank (Mantel-Cox) test; N = 40/20/43/4 for wound/RCI 8.5/9.0/9.5 Gy.

We previously demonstrated that 9.5 Gy RCI (TBI + SW) significantly augments the risk of morbidity and mortality compared to 9.5 Gy RI (TBI alone) [4,21]. To further determine the detrimental effects of RCI, we examined the effects of RCI at different doses of TBI (8.5/9.0/9.5 Gy) on 30-day lethality, body weight loss, and wound closure delay in an established RCI mouse model using female B6D2F1/J mice (13–15 weeks old), as described in the Material and Method section (N = 80/20/80/60 for wound/RCI 8.5/9.0/9.5 Gy). The Kaplan-Meier survival curve (Figure 1A) demonstrated that the 30-day survival after RCI was radiation dose-dependent. All animals in the SW and 8.5 Gy RCI groups survived for 30 days. Therefore, 8.5 Gy is a sublethal dose for RCI. The percentage of survival on day 30 following the lethal doses of RCI (9.0 or 9.5 Gy) and the median survival time (shown in parentheses) were 51.25% (>30 days) for 9.0 Gy and 33.33% (15.5 days) for 9.5 Gy. The difference in the percentage of the 30-day survival is significant for 9.0 Gy vs. 8.5 Gy/SW alone and 9.5 Gy vs. 8.5 Gy/SW alone (Figure 1A). In addition, body weights (Figure 1B) were measured; wound images (Figure 1C) were taken from all groups of mice at indicated time points, and the wound sizes (Figure 1D) were measured on days 1, 3, 7, 14, 21, and 28 after TBI. Wounds were not debrided and scabs fell off naturally. In some cases, scab diameters were considered to be the wound diameter. Time to wound closure was also recorded (Figure 1E). Our data demonstrated that RCI induced body weight loss (decrease in % body weight from day 0) and wound closure delay (decrease in % wound closure on day 1 and increase in wound closure time) in a radiation dose-dependent fashion. Furthermore, data in Figure 1B shows that, unlike mice in the SW alone and sublethal (8.5 Gy) RCI groups, mice in the lethal dose RCI groups (9.0 or 9.5 Gy) never fully recovered from the RCI-induced body weight loss. Figure 1D shows that the wound size in the 8.5 Gy RCI group is almost overlapping with that in the SW alone group, indicating that the sublethal RCI delayed the wound healing but closed it at a same time as the SW alone took, whereas lethal RCI significantly delayed wound closure compared to SW alone and 8.5 Gy RCI groups at each time point. Figure 1E shows that, on day 30 after TBI, all wounds from SW alone and 8.5 Gy RCI groups and 90% of wounds from the 9.0 Gy RCI group were closed, but all wounds from the 9.5 Gy group were still open. The median wound closure time is 13 days for SW, 16 days for 8.5 Gy RCI, 22 days for 9.0 Gy RCI, and >30 days for 9.5 Gy RCI.

### 3.2. RCI Significantly Increases NEU, MONO, and NEU/LYM and Decreases LYM and PLT

**Figure 2:**
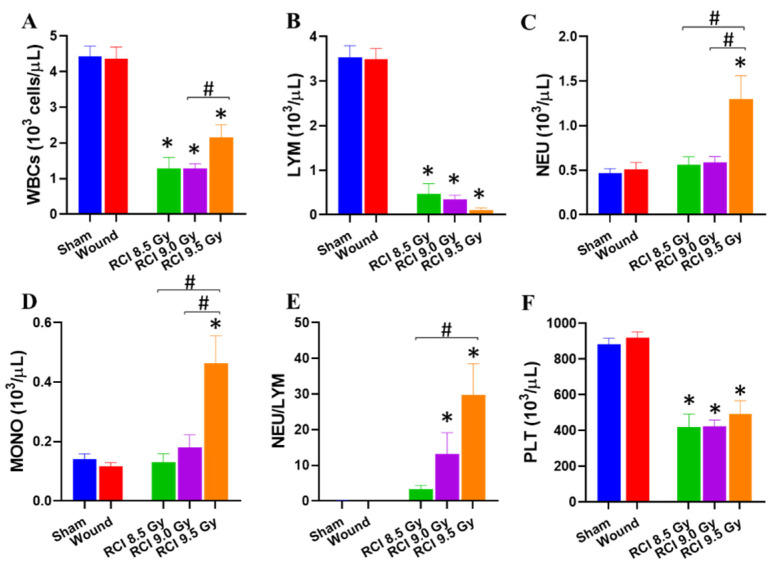
Neutrophil (NEU), monocyte (MONO), and neutrophil/lymphocyte ratios (NEU/LYM) are elevated and LYM and platelets are declined in lethal doses of RCI mice. (**A**,**B**,**F**) White blood cells (WBCs), lymphocytes (LYM), and platelets (PLT) were significantly decreased in all RCI groups. However, neutrophils (NEU) and monocytes (MONO) were significantly elevated in 9.5 Gy RCI (**C**,**D**). The NEU/LYM ratio was significantly elevated in 9.0/9.5 Gy RCI (**E**). * *p* < 0.05 for RCI vs. sham by one-way ANOVA and Dunnett’s multiple comparisons test; # *p* < 0.05 for comparison among three RCI groups by one-way ANOVA and Tukey’s multiple comparisons test; N = 10–35.

Lethal dose RCI delays wound closure, and then an open wound increases the chance of infection. The complete blood cell count (CBC) was measured. The day 30 CBC data demonstrated that white blood cells (WBCs, Figure 2A) and lymphocytes (LYM, Figure 2B) were significantly decreased in all RCI groups, while neutrophils (NEU, Figure 2C) and monocytes (MONO, Figure 2D) were increased after 9.5 Gy RCI. The NEU/LYM ratio was significantly elevated in RCI mice at 9.0 Gy and 9.5 Gy (Figure 2E).

Platelet (PLT, Figure 2F) counts were significantly declined in three RCI groups compared to the sham-irradiated group.

### 3.3. RCI at 9.5 Gy Induces a Systemic Proinflammatory Response in Mice

**Figure 3:**
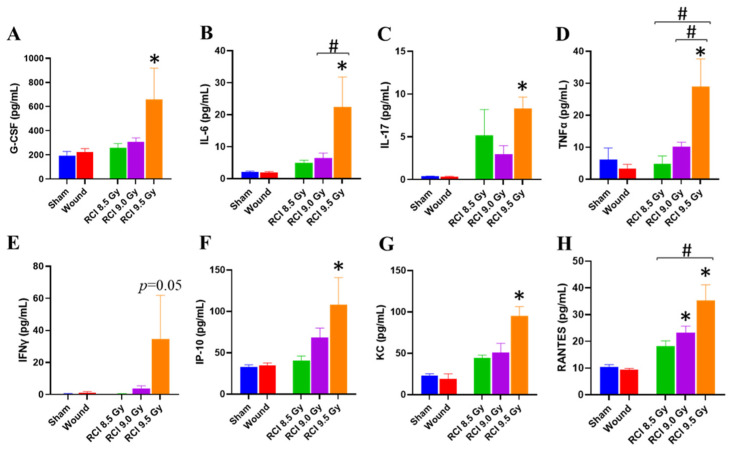
Increased levels of selected proinflammatory cytokines and chemokines are observed in 9.5 Gy RCI mice. (**A**) G-CSF (granulocyte-colony stimulating factor); (**B**) IL-6 (Interleukin 6); (**C**) IL-17 (Interleukin 17); (**D**) TNF-α (Tumor necrosis factor alpha); (**E**) IFNγ (Interferon-gamma); (**F**) IP-10 (interferon-gamma-induced protein 10); (**G**) KC (keratinocyte-derived cytokine); (**H**) RANTES (regulated upon activation, normal T cell expressed, and secreted). * *p* < 0.05 for RCI vs. sham by one-way ANOVA and Dunnett’s multiple comparisons test; # *p* < 0.05 for comparison among three RCI groups by one-way ANOVA and Tukey’s multiple comparisons test; N = 3–7.

Radiation exposure induces the cytokine cascade activation to modulate the immune system [33,34]. Next, we investigated whether the RCI modulated cytokine production in the surviving RCI mice exposed to the lethal doses. Therefore, the levels of 44 cytokines and chemokines in mouse serum samples collected on day 30 after irradiation from surviving animals in all groups were determined. Among these cytokines and chemokines, an aberrant proinflammatory cytokine/chemokine profile in Figure 3, shown as increased levels of proinflammatory cytokines (G-CSF, IFNγ, IL-6, IL-17, TNFα, Figure 3A–E) and chemokines (IP-10, KC, RANTES [regulated upon activation, normal T cell expressed and presumably secreted], Figure 3F–H), was observed in 9.5 Gy RCI mice. Five pro-inflammatory cytokines are involved in producing granulocytes, recruiting NEU and MONO to the wound site, or increasing NEU and MONO function; and the three elevated chemokines have been implicated in inflammatory diseases [35,36,37,38]. In 9.0 Gy RCI mice, only RANTES was elevated in comparison with the sham-irradiated mice (Figure 3H) and no differences were observed between 8.5 Gy RCI mice and SW/sham-irradiated animals (Figure 3).

### 3.4. RCI with Doses Ranging from 8.5 Gy to 9.5 Gy Induces Hypocellularity to a Similar Degree of Severity in Mouse Bone Marrow (BM) on Day 30 after RCI

**Figure 4:**
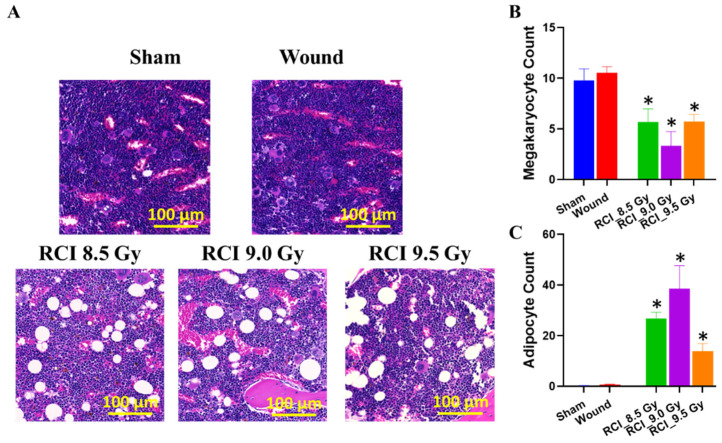
Bone marrow hypocellularity is induced by RCI at different doses. (**A**) Representative images of histological sections of sternum obtained on day 30 post-TBI stained with hematoxylin and eosin (H&E), scale bar: 100 µm. (**B**,**C**) Quantification of megakaryocyte and adipocyte. * *p* < 0.05 for RCI vs. sham by one-way ANOVA and Dunnett’s multiple comparisons test; N = 4–13.

Vegesna et al. examined the impact of local or systemic irradiation on skin wound healing in mice, and results from that study suggest that the degree of hematopoietic suppression determines the healing response in irradiated skin wounds [27]. Therefore, we examined the BM damage induced by RCI at different doses of radiation exposure (8.5/9.0/9.5 Gy). Histological sections of sternum with hematoxylin and eosin (H&E) obtained on day 30 after TBI demonstrated that, compared to the sham-irradiated and SW alone, RCI caused an apparent hypocellularity in the mouse BM (Figure 4A). Quantitatively, RCI significantly decreased the production in megakaryocytes (Figure 4B). As a consequence, the marrow was replaced by adipocytes (Figure 4C) in the RCI groups, since an increase in the adipocyte number in BM is secondary to a decrease in hematopoietic cells [21,39,40,41,42]. Histological images (Figure 4A) demonstrated that the RCI with doses ranging from 8.5 Gy to 9.5 Gy induced the BM hypocellularity to a similar degree of severity, which was confirmed by no apparent dose responses in the quantification of megakaryocytes and adipocytes (Figure 4B,C).

### 3.5. Chemokine Levels Are Elevated in Skin Wound Sites of RCI Mice Exposed to 9 Gy

**Figure 5:**
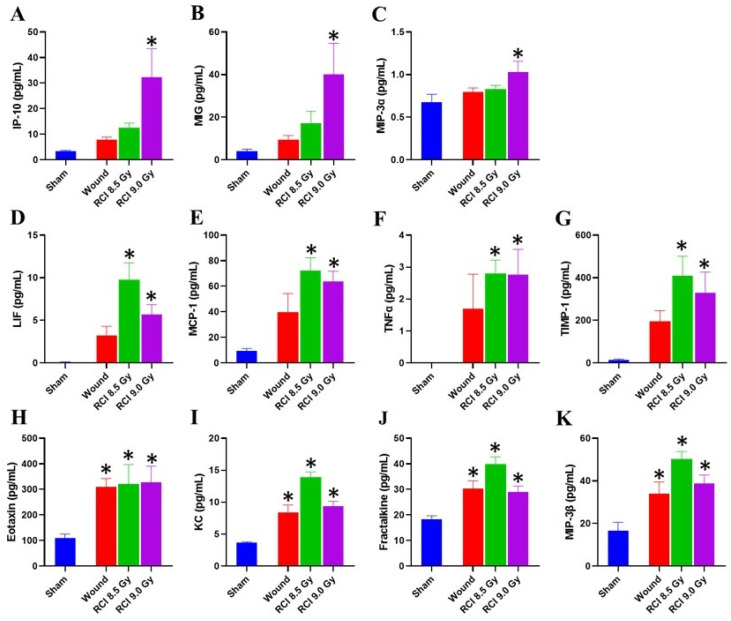
Chemokine levels are elevated in the skin at wound sites of 9.0 Gy RCI mice. (**A**–**C**) Three chemokines IP-10, MIG, and MIP-3α were significantly elevated in skin at wound sites of 9.0 Gy RCI mice. (**D**–**G**) Four chemokines LIF, MCP-1, TNFα, and TIMP-1 were increased in skin at wound sites of RCI groups. (**H**–**K**) Other four chemokines eotaxin, KC, fractalkine, and MIP-3β were elevated in skin at wound sites of both wound and RCI groups. * *p* < 0.05 for wound or RCI vs. sham by one-way ANOVA and Dunnett’s multiple comparisons test; N = 4.

As mentioned above, RCI did not result in bone marrow damage in a dose-dependent manner; however, the delay in wound healing induced by RCI was displayed in a radiation dose dependent manner, which indicates that the hematopoietic suppression does not determine the wound healing response in RCI mice. The next question was whether local factors were responsible for the delayed wound healing. Cytokines and chemokines in wound sites have been identified to mediate the healing response [43]; therefore, the day 30 skin tissue lysates prepared from wounds in surviving animals were studied using a cytokine/chemokine array. The skin tissue cytokine/chemokine profile, as shown in Figure 5, is different from the serum profile (Figure 3). Compared to the sham-irradiated mice, three chemokines, IP-10, MIG, and MIP-3α (Figure 5A–C), were significantly elevated in samples from wound sites of RCI mice exposed to 9.0 Gy. In addition, the LIF, MCP-1, TNFα, and TIMP-1 (Figure 5D–G) are significantly elevated in skin wounds of 8.5/9.0 Gy RCI mice but not of the SW alone mice. Furthermore, eotaxin, KC, fractalkine, and MIP-3β (Figure 5H–K) were increased in all skin wounds, but not in a radiation dose-dependent manner.

### 3.6. Sublethal RCI and Wounding alone, but Not Lethal RCI, Upregulate VEGF Expression in Skin Wound Sites on Day 30 after TBI

**Figure 6:**
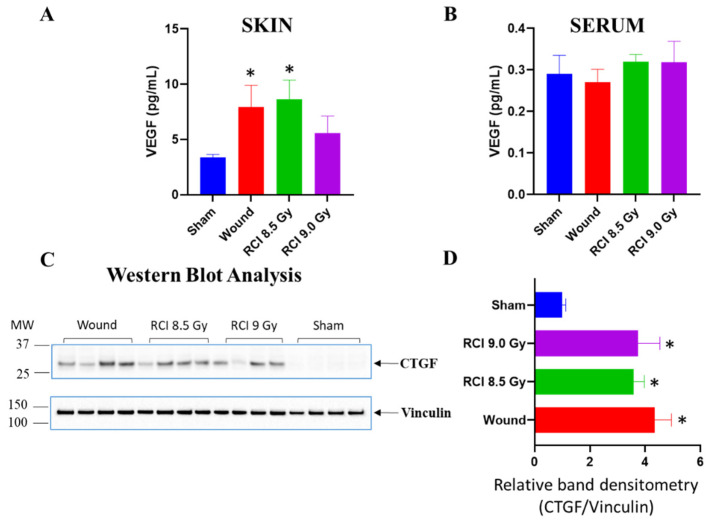
Both 8.5 Gy RCI and SW alone significantly upregulate VEGF expression in skin wounds on day 30, and no significant differences are observed between sham and 9.0 Gy RCI (**A**). (**B**) There were no differences in serum VEGF levels in all groups. (**C**) Western blot analysis of CTGF expression in skin wounds, and their band densitometry analysis as shown in (**D**). CTGF (connective tissue growth factor) expression was upregulated in wound and RCI groups. * *p* < 0.05 for wound vs. sham, RCI 8.5 Gy vs. sham, and RCI 9.0 Gy vs. sham by Mann–Whitney Test (unpaired, two-tailed); N = 4. MW: molecular weight.

It is evident that growth factors (VEGF, PDGF, FGF, TGF, CTGF, etc.) secreted by endothelial/epithelial cells, fibroblasts, platelets, or leukocytes/macrophages influence the wound healing process via endocrine, autocrine, or paracrine mechanisms [44,45]. In response to the tissue injury and/or hypoxia secondary to metabolic dysfunction, the release of VEGF into the wound microenvironment is stimulated to improve wound healing [46,47,48]. The down-regulation of VEGF has been implicated in non-healing acute and chronic wounds [45,49]. Thus, we assessed the level of VEGF expression in skin samples in wound sites on day 30 after TBI. In comparison with sham-irradiated samples, both 8.5 Gy RCI and SW alone mice significantly upregulated VEGF expression in skin wounds on day 30 (Figure 6A). No significant differences were observed between sham-irradiated and 9.0 Gy RCI groups (Figure 6A). There were no differences in serum VEGF level in all groups (Figure 6B). CTGF is implicated in early wound repair [50], and has been demonstrated to regulate various biological processes via modulating the activities of several cytokines and growth factors [51]. In skin lysates prepared from day 30 skin wounds, Western blot data demonstrated that the CTGF expression was significantly upregulated in SW alone, and in 8.5 Gy/9.0 Gy RCI groups, compared to the sham-irradiated group (Figure 6C,D), suggesting CTGF is a potential biomarker for skin wounding.

### 3.7. RCI Mice Display Dysregulated Collagen Homeostasis in Skin Wound Scar

**Figure 7:**
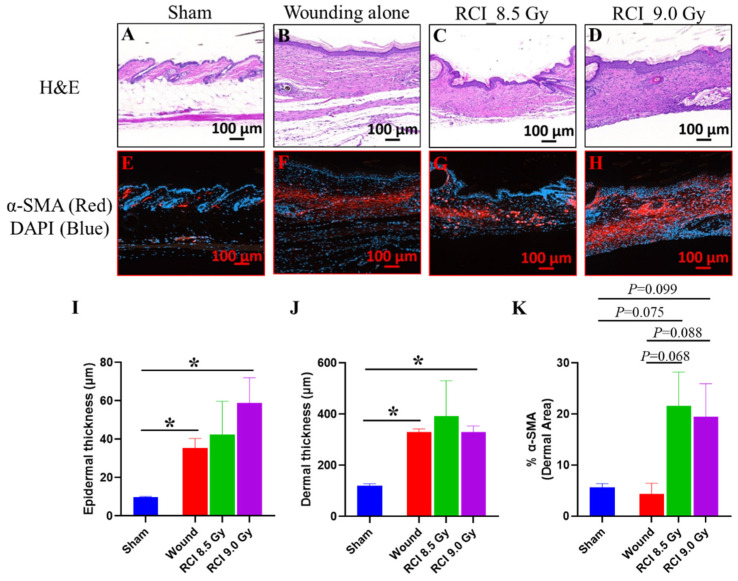
Histological and immunohistochemical (IHC) evaluation of skin wounds to assess the epidermal and dermal responses after SW alone or 8.5 Gy/9.0 Gy RCI. (**A**–**H**) Representative images of histological sections of skin wound scar day 30 after TBI stained with hematoxylin and eosin (H&E) (**A**–**D**). (**E**–**H**) Representative images of IHC-stained skin wound scar sections for alpha-smooth muscle actin (α-SMA, a marker of myofibroblast, red) and nuclei (4′, 6-diamidino-2-phenylindole, DAPI, nuclear DNA, blue). Scale bar: 100 µm. (**I**–**K**) Quantification of epidermis thickness (µm), dermis thickness (µm), and dermal α-SMA positive area (%). * *p* < 0.05, wound or RCI vs. sham by unpaired *t*-test (two-tailed); N = 3/group.

**Figure 8:**
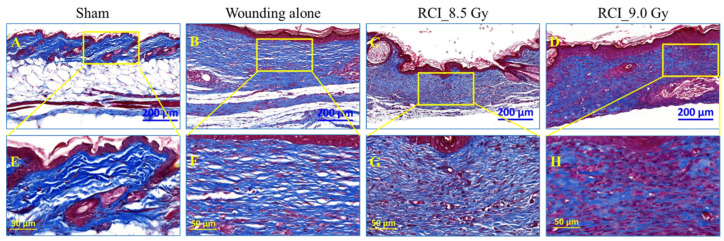
TBI perturbs collagen deposition during wound healing. Collagen morphology/organization in scars of skin wounds was evaluated by Masson’s trichrome staining (Blue: collagen; Red: cytoplasm; Black: nuclei). (**A**–**D**) Representative images of histological sections of skin wound scar day 30 after TBI (low magnification) from sham, SW alone, RCI at 8.5 Gy, and RCI at 9.0 Gy groups, N = 3, scale bar: 200 µm. (**E**–**H**) High magnification images of respective insets displayed in (**A**–**D**), N = 3/group, scale bar: 50 µm.

We further performed the histological and immunohistochemical (IHC) evaluation of skin wounds to assess the epidermal and dermal responses after SW alone or RCI. In Figure 7, the histological sections of skin wounds obtained on day 30 after TBI stained with H&E were examined. The images and quantification data displayed a significant increase in the epidermal and dermal thickness (Figure 7B,D,I,J) in the scars of SW alone and 9.0 Gy RCI groups in comparison with normal skin from sham-irradiated group (Figure 7A). More variation was observed in the 8.5 Gy RCI group than in the SW alone group (Figure 7C,I,J). In normal and chronic wound repair, myofibroblasts are the main extracellular matrix of (ECM)-secreting cells [52]. Since ECM plays essential roles in the regulation of wound healing [53], we next estimated the activity of the myofibroblasts by IHC staining for its marker, the alpha-smooth muscle actin (α-SMA). Different from the H&E staining, the immunoreactivity of the α-SMA in the scar dermis was not significantly changed but with a higher trend in skin wounds of 8.5/9.0 Gy RCI mice, when compared to either sham-irradiated or SW alone mice (Figure 7E–H,K). Collagen is an important component of ECM and is critical in regulating the wound healing process [53]. To evaluate the collagen morphology/organization in scars of skin wounds, Masson’s trichrome staining was performed and the image data from representative mouse samples in different groups are shown in Figure 8. In normal skin from the sham-irradiated group, collagen was tightly packed and orderly organized in a net-like structure (Figure 8A,E). Whereas, in the scar from SW alone group, the collagen was loosely packed and aligned parallelly to the basement membrane of skin (Figure 8B,F). More importantly, we found that the TBI perturbed the collagen deposition during wound healing. In scars from RCI at 8.5 Gy and 9.0 Gy groups, the collagen was randomly placed. In contrast to the distinct parallel collagen alignment found in the wound from the SW alone group, the collagen morphology in wounds from RCI groups was irregular, patchy, and clustered (Figure 8C,D,G,H).

## 4. Discussion

The results from the current study demonstrated, for the first time, that the delayed wound closure in RCI mice is not specifically dependent on the extent of hematopoietic suppression, but is significantly impacted by systemic and local factors, including cytokines/chemokines, growth factors, and dysregulated collagen homeostasis in the wounded area.

First, we demonstrated that, in the RCI mouse model (TBI + SW), lethal doses TBI at 9.0 Gy or 9.5 Gy resulted in delayed wound healing in a radiation dose-dependent manner, indicating that the toxicity of radiation impacted the skin wound recovery in mice. Furthermore, our data imply that the delay in wound healing is closely associated with the increased morbidity (body weight loss) and mortality (30-day lethality) in RCI mice. Radiation-caused body weight loss is one of the severe clinical manifestations of sickness. Radiation combined with skin wound delays the wound healing and further increases the risk of mortality due to the nutrition and energy loss, immune system disorder, and systemic and wound area inflammation, implying the increased chances of infection. Results in Figure 1C,D, and E demonstrated that the wound closure was significantly delayed by lethal doses of TBI in mice and no wound closure was observed in the 9.5 Gy RCI group by 30 days post-TBI. In contrast, the wound was completely closed by day 13–16 in SW alone and the 8.5 Gy sublethal RCI groups. Results from our previous RCI (9.5 Gy TBI + SW) mouse studies demonstrated that the compounds (e.g., ghrelin [17], ciprofloxacin [10], BMSCs [12], or L-citrulline [21]) showing the effects on improving wound healing and body weight have the mitigative efficacy against RCI. In addition, Zawaski, et al. reported that wounds in RCI mice (circular wound of diameter of 19 mm in combination with 6 Gy TBI) receiving topical antibiotics healed faster and had a higher survival rate than the vehicle-treated mice [54]. Thus, there is increasing evidence that interventions that could improve wound healing and relief from body weight loss hold great promise for mitigating the RCI-induced mortality.

The accumulating reports demonstrate that traumatic injury, including skin wounding, relevant to the lethal radiation-induced ARS, could increase the mortality [4,5,6,8,9,13,21]. Increased susceptibility to infection has been proposed to contribute to the synergism between skin wounding and lethal radiation exposure [4,8,9]. The current study also provides solid evidence, demonstrating that a systemic proinflammatory status is detectable in the 30-day surviving mice after the RCI in a radiation dose-dependent manner and 9.5 Gy RCI was manifested: 1. Increased neutrophil and monocyte counts; 2. An elevated neutrophil to lymphocyte ratio; 3. An increased level of proinflammatory cytokines/chemokines in serum (e.g., G-CSF, IL-6, IL-17, TNFα, IFNγ, IP-10, KC, RANTES), when compared to sham-irradiated animals. Whereas, we only observed the increase in the neutrophil to lymphocyte ratio and serum RANTES level in 9.0 Gy RCI mice, and no significant difference was observed between 8.5 Gy RCI and sham-irradiated groups. The similar pattern of systemic proinflammatory state and wound closure in RCI mice explains that lethal RCI delays wound closure, and an open wound increases the chance for infection. Ledney et al. at AFRRI conducted a series of studies to determine the relative incidence and species of bacteria on the wound and in the liver of RCI, RI, and SW mice, and demonstrated that the gram-positive bacteria detected in the liver were similar to those bacteria colonizing the skin wound site, indicating that the bacterial sepsis causing animal death in the RCI group was partially from the wound areas [19]. Thus, the prolonged and elevated inflammation could further cause wound area infection and retard wound healing. Therefore, it is important to identify the factors interfering with wound healing in RCI animals.

With such a complicated biological process, wound healing is achieved via four distinct but conjoining phases: hemostasis, inflammation, proliferation, and remodeling. Systemic and/or local factors can interfere with the right sequence and time frame of this process, thus impairing wound healing [25]. The evidence of inflammation including the increased neutropenia and monocytes in periphery blood and proinflammatory factors in the serum and wound area up to 30 days after 9.5 Gy RCI may interrupt the normal skin wound healing process and eventually resulted in no wound closure in these animals. The bone marrow supplies endothelial progenitor cells (EPCs) and inflammatory cells to the wound site, where they control the migration and proliferation of epithelial cells and dermal mesenchymal cells during the inflammation phase [55,56]. In this study, we found that, despite the finding that hematopoietic suppression can be detected in the 30-day surviving RCI animals, it was induced by 8.5/9.0/9.5 Gy RCI at the similar level of severity and no apparent dose responses in the quantification of megakaryocytes and adipocytes, as shown in Figure 4. This is consistent with our recently published study [21]. In that study, we reported that 30-day surviving mice after 9.5 Gy RCI displayed a mild BM hypocellularity compared to mice receiving 9.5 Gy RI alone, and treatment with PEG-G-CSF did not further improve the BM cellularity in RCI mice [21]. Using the adipocyte number as a parameter to measure the BM hematopoietic cell loss has been well accepted because the induced adipocytes come from its precursor mesenchymal stem cells (MSCs) in BM. The radiation-induced marrow cellular senescence mediates MSCs going through the lineage switching toward an adipogenic lineage and results in increased adipocyte counts in BM [39,40,41]. We postulate that the reduced sensitivity of the hematopoietic system in RCI is due to the synergistic effect induced between radiation and wound trauma, which agrees with the conclusion reached by Ledney et al.; the trauma increased the hematopoietic proliferative compartments of RCI mice [19]. Since RCI induces a radiation dose-dependent delay in wound healing, but not hematopoietic suppression, it suggests that hematopoietic suppression alone neither determines the healing response of wounds nor contributes to an increased risk of mortality in RCI mice. Wound healing is also influenced by skin cells, the action of local proteins, and glycoproteins to the wound itself, such as growth factors, cytokines, chemokines, and their receptors or inhibitors [57]. For example, data from the current study indicated that the altered levels of chemokines (such as IP-10, MIG, MIP-3α) and growth factors (e.g., VEGF), and dysregulated collagen homeostasis are implicated in the lethal RCI-induced delay in wound closure. Our results are different from the data demonstrated by an early study designed to evaluate the impact of the local or systemic irradiation on the healing of full-thickness skin wounds in mice [27]. In that study, the endpoint was the wound tensile strength assessed on day 14 after irradiation. Based on the histological observation, there were significantly less inflammatory cells in the wounds from TBI compared with those receiving only local irradiation; the study concluded that the extent of hematopoietic suppression determines the healing response in irradiated skin [27]. We think the discrepancy is due to the different endpoint and the assessment time between these two studies.

It is worth noting that one limitation of this study is that we only evaluated the hematopoietic damage and local factors (cytokines/chemokines, growth factors, and histological assessment of skin wound) using samples collected on day 30 after TBI due to the restriction of our project aims. One of the main goals of the study was to determine the association of delayed skin wound healing with the mortality of RCI animals. Since the mortality of RCI mice is usually assessed in a 30-day survival study, our initial attempt to link the delayed healing to a higher mortality was to observe and report the alterations in mouse skin tissue harvested on day 30 post-TBI. We also acknowledge that only looking at endpoints in surviving animals will not tell the entire story, because the decedents or the moribund animals were excluded due to the current approved IACUC protocol. It would be more relevant if we could also look in decedents. Nonetheless, for lethal doses of RCI, the mice found moribund must be euthanized, but the matched animals in either the sham or skin wounding alone groups were not available to serve as a control for comparison in this case. As aforementioned, the wound healing occurs in four phases. The examination of the influence of these factors on wound repair at early time points, such as days 1, 3, 7, 14, and 21 after TBI, would provide us a better understanding of the mechanisms underlying the delayed wound healing at all phases of the wound healing cascade in RCI animals. Our future research is aimed at identifying the potential molecular targets and significantly involved pathways associated with the RCI-induced wound healing delay at different time points in different sexes of animals. We believe that the advanced understanding of mechanisms for the impaired wound healing in both sexes of mice would lead to effective therapeutics that accelerate acute wound healing and save lives.

The skin tissues samples from 9.5 Gy RCI animals were not included for some endpoints, because we initially experienced some technical difficulty when collecting skin tissues from the 9.5 Gy RCI mice on day 30 post-TBI. Since our data presented in Figure 1A demonstrate that both 9.0 Gy and 9.5 Gy are lethal doses and show that there were no significant differences in 30-day lethality observed between these two lethal doses, we then focused on comparing the 9.0 Gy lethal dose vs. 8.5 Gy sublethal dose vs. skin wounding alone for the following endpoints: cytokine/chemokine level in wounded skin lysates, growth factor expression in wounded skin, skin histology, and immunohistochemistry.

Taken together, the results from this study demonstrated that the toxicity of radiation plays an important role in the induction of the inflammatory response systemically and at wounded areas, a delay of skin-wound healing, and increases in animal mortality in a radiation dose-dependent manner. The delayed wound closure in RCI mice is not specifically dependent on the extent of hematopoietic suppression, but rather, is significantly impacted by systemic inflammation and local factors including cytokines/chemokines, growth factors, and dysregulated collagen homeostasis in the wounded area.

## Data Availability

Not applicable.
